# Current practice in the perioperative management of patients with diabetes mellitus: a narrative review

**DOI:** 10.1016/j.bja.2023.02.039

**Published:** 2023-04-13

**Authors:** Kieran Crowley, Pádraig Ó Scanaill, Jeroen Hermanides, Donal J. Buggy

**Affiliations:** 1 Department of Anaesthesiology & Perioperative Medicine, Mater University Hospital, School of Medicine, University College Dublin, Dublin, Ireland; 2 Academic Medical Centre, Amsterdam, the Netherlands; 3 Outcomes Research Cleveland Clinic, Cleveland, OH, USA

**Keywords:** diabetes mellitus, insulin, oral hypoglycaemic agent, postoperative complications, surgery

## Abstract

The prevalence of diabetes is increasing, and patients with diabetes mellitus have both an increased likelihood of requiring surgery and of developing postoperative complications when they do. We summarise available evidence underpinning current guidelines on preoperative assessment and optimisation, perioperative management of prescribed insulin and oral hypoglycaemic medication, intraoperative glycaemic control, and postoperative patient care.

**Editor's key points**

• The prevalence of diabetes mellitus is increasing, but evidence for many decisions important in optimal perioperative management is lacking.
• This narrative review summarises current evidence underpinning updated guidelines.
• Patients with type 2 diabetes mellitus (T2DM) undergoing elective surgery should continue their metformin, GLP-1RA analogues, and DPP-4 inhibitors including on the day of surgery, but SGLT-2 inhibitors should be stopped 3 days before.
• Large observational studies and trials are needed in patients with diabetes mellitus undergoing surgery to define optimal perioperative management for early and intermediate postoperative outcomes.

Diabetes mellitus (DM) is a chronic multisystem disease which is becoming increasingly prevalent in the general population. It is estimated that approximately 537 million people worldwide have DM. Projections are that this may increase to 700 million by 2045. In Europe, approximately 1 in 11 adults have DM – equating to roughly 61 million individuals.^1^

It has been shown in numerous studies that patients with DM undergo surgical procedures more often than patients without it.^2^^,^^3^ A study suggests that one in four patients undergoing surgery has a chronic disease – which has an associated 10-fold increase in postoperative death.^4^ It analysed data for more than 8 million patients and found that 8% had diabetes – making it the second most common perioperative comorbidity.^4^ A separate study suggests that patients with diabetes account for 15% of all operative procedures,^5^ placing a major burden on healthcare systems as these patients are clinically more complex.^6^

Perioperative management of the patient with diabetes is an often under-emphasised clinical challenge, even though it is a vulnerable time for this already higher-risk patient cohort. Unpredictable preoperative fasting times, potentially hazardous administration of intravenous medications including insulin, and the stress response of surgery may lead to adverse postoperative outcomes.^2^^,^^7^ Besides the human cost, there are also economic consequences including increased hospital length of stay.^2^

There are numerous guidelines available for the perioperative management of DM.^5^^,^^8^ These are based largely on expert opinion and consensus from best practice panels, which sometimes results in differing clinical practice between centres at a regional level and internationally. Discrepancies between these different guidelines reflect both the scarcity of available evidence in perioperative management of the patient with diabetes, and the use of outdated guidelines. This narrative review aims to summarise current data on how perioperative management of patients with diabetes may influence clinical outcomes and highlight priorities for future original investigation for this important, and often neglected, clinical cohort.

## Methods

We searched databases including PubMed, MEDLINE, and EMBASE for all types of articles in the English language. We used several keywords and combinations of keywords including diabetes, perioperative management, perioperative care, perioperative outcomes, surgery, emergency surgery, surgical outcomes, anaesthesia, anaesthetics, and postoperative outcomes. These keywords were limited to either ‘Title’ or ‘Title/abstract’. Our Boolean search strategy is shown in Supplementary Appendix 1.

### Preoperative care

#### Type of diabetes mellitus

Preoperative assessment of the patient with diabetes mellitus comprises several requirements. The type of diabetes mellitus must be ascertained. Traditionally this is classified (as shown in Table 1) into type 1 diabetes mellitus (T1DM; absolute insulin deficiency, DM 1), T2DM (peripheral insulin resistance and inadequate insulin secretion, DM 2), gestational diabetes, and specific types of diabetes attributable to other causes.^9^ A recent scoping review found that the definition of DM is variable between different studies.^10^ Not only is DM defined in different ways, but definitions for glycaemic control are also not uniformly defined.^10^ This makes it difficult to compare evidence between different studies.

**Table 1:** Classification of diabetes mellitus. MODY, maturity-onset diabetes of the young.

1. Type 1 diabetes mellitus (caused by β-cell destruction – therefore absolute insulin deficiency) (a) Autoimmune (b) Idiopathic
2. Type 2 diabetes mellitus (owing to a combination of peripheral insulin resistance and insulin deficiency)
3. Other A. Monogenic diabetes syndromes – MODY, neonatal diabetes B. Diseases of the exocrine pancreas – Pancreatitis, neoplasm, cystic fibrosis, pancreatectomy C. Endocrinopathies – Cushing's syndrome, acromegaly, phaeochromocytoma D. Drug or chemical induced – Thiazides, glucocorticoids, nicotinic acid, β-adrenergic agonists E. Infections – Cytomegalovirus, congenital rubella F. Genetic syndromes associated with diabetes mellitus – Down's syndrome, Klinefelter's syndrome, myotonic dystrophy, Friedreich's ataxia
4. Gestational diabetes

Often, hospitals poorly differentiate patients with T1DM from patients with T2DM. A retrospective cross-sectional study (*n*=2259) demonstrated that if similar perioperative treatment is provided to both T1DM and T2DM patients, those with T1DM will have poorer glycaemic control.^11^ It also showed that patients with T1DM had a higher perioperative peak glucose concentration (11.0 [8.2–14.7] *vs* 9.4 [7.7–11.7], *P*<0.001)^11^ and a higher incidence of perioperative hyperglycaemia compared with T2DM patients (63% *vs* 43%, *P*<0.001), along with more frequent episodes of hypoglycaemia (7.1% *vs* 1.3%, *P*<0.001).^11^ It is clear from studies such as this that the different physiology and disease process between T1DM and T2DM is poorly appreciated at a clinical level and that more needs to be done to establish clear perioperative pathways to safely manage these patients.

Even though the incidence of gestational and other types of diabetes is much lower than T2DM and T1DM, it is important to recognise the actual diagnosis. For example, patients with pancreatogenic diabetes are more unstable in the perioperative period as compared with gestational or glucocorticoid induced diabetes.^12^

#### Non-insulin medication

The second component of preoperative assessment is patients' current medication. These may be classified as insulin and non-insulin agents (Table 2). There are various conflicting guidelines as to whether injectable and oral glucose-lowering agents should be continued preoperatively, reflecting a lack of evidence addressing this clinical question.^5^ The well-known risks with some of these agents is that they may cause hypoglycaemia or diabetic ketoacidosis. Therefore, preoperative review of the patients' medication by a pharmacist may be of benefit to reduce medication errors.^13^

**Table 2:** A comparison of guidelines from professional societies of key interventions in perioperative management of patients with diabetes mellitus. ADA, American Diabetes Association; BD, twice daily; BGL, blood glucose level; CPOC, Centre for Perioperative Care; CSII, continuous subcutaneous insulin infusion; DPP-4, dipeptidyl peptidase-4; DrEaMing, drinking, eating, and mobilising; ERAS, enhanced recovery after surgery; GKI, glucose–potassium–insulin; GLP-1RA, glucagon-like peptide 1 receptor agonist; HbA1c, glycated haemoglobin; OD, once daily; SGLT-2, sodium-glucose cotransporter 2; TDS, three times daily; VRIII, variable rate intravenous insulin infusion.

Intervention	CPOC^5^	AAGBI^8^	ADA^66^
Insulin			
Once daily long-acting insulin – morning dose	Day before surgery – normal dose.Day of surgery – give 80% of dose.	Day before surgery – reduce dose by 20%.Day of surgery – reduce dose by 20%.	Day of surgery – give 75–80% of normal dose.
Once daily long-acting insulin – lunchtime dose	Day before surgery – give 80% of dose.Day of surgery – restart insulin at normal dose once eating and drinking.	Nil.	
Once daily long-acting insulin – evening dose	Day before surgery – give 80% of dose.Day of surgery – no adjustment required.	Day before surgery – reduce dose by 20%.Day of surgery – check BGL on admission.	
×2 daily long-acting insulin	Evening before surgery – give 80% of dose. Day of surgery – give 80% of morning dose. Evening dose unchanged.	Day before surgery – no dose change. Day of surgery – halve the morning dose. Evening dose unchanged.	
×2 daily premixed insulin	Day before surgery – no adjustment required. Day of surgery – give 50% of usual dose.	Day before surgery – no adjustment required. Day of surgery – give 50% of usual dose in the morning as intermediate-acting insulin.	Day of surgery – give 50% of usual dose.
×3 premixed insulin	Day before surgery – no adjustment required. Day of surgery – halve usual morning dose. Omit lunchtime dose.	Day before surgery – no adjustment required. Day of surgery – halve morning dose. Omit lunchtime dose.	Day of surgery – give 50% of usual dose.
Short-acting insulin with meals	Day before surgery – no adjustment required.Day of surgery – omit dose if no meal eaten. If not eating, consider basal dose.	Day before surgery – no adjustment required.Day of surgery – omit dose if no meal eaten.	
Metformin	Day before surgery – take as normal. Day of surgery: If BD dosing, take as normal. If TDS dosing, omit lunchtime dose.	Day before surgery – take as normal. Day of surgery – take as normal.	Withhold on day of surgery.
Sulphonylureas	Day before surgery – take as normal. Day of surgery – omit morning dose.	Day before surgery – take as normal. Day of surgery – omit morning dose.	Day of surgery – omit morning dose.
GLP-1RA	Take as normal.	Take as normal.	No data
DPP-4 inhibitors	Take as normal.	Take as normal.	No data
SGLT-2 inhibitors	Omit day before surgery and day of surgery.Check capillary blood ketones daily.	Day before surgery – take as normal.Day of surgery – halve the usual morning dose.Omit the day after surgery.	Discontinue 3–4 days before surgery.
Preoperative fasting time	Minimise.	Minimise.	
Priority on list	Promote day surgery.	Patient should be first on the operating list. Day of surgery admission.	
HbA1c	Defer + optimise elective case if >69 mmol mol^−1^.	Defer + optimise elective case if >69 mmol mol^−1^	
Glycaemic control	Between 6 and 10 mmol L^−1^ (6–12 is acceptable).	Between 6 and 10 mmol L^−1^ (6–12 is acceptable).	Between 4.4 and 10.0 mmol L^−1^
VRIII *vs* GKI *vs* none	VRIII recommended – BGL target range of 6–12 mmol L^−1^. Continue basal insulin at 80% of normal dose while on VRIII.	VRIII recommended. Continue basal insulin at 80% of normal dose while on VRIII.	Basal bolus
Intravenous fluid	5% dextrose in 0.45% saline with 0.15%/0.3% potassium chloride – while on VRIII.	5% glucose in 0.45% saline with 0.15%/0.3% potassium chloride – while on VRIII.	
CSII	Continue during perioperative period when possible.	Continue if only missing 1 meal.	
Postoperative management	Encourage early DrEaMing.	ERAS strategies.	
Ketone measurement	Check if BGL > 13 mmol L^−1^ on 2 occasions **or** patient becomes unwell. Measure capillary blood ketones daily if patient is normally on SGLT2 inhibitors	Check if BGL >12 mmol L^−1^ and insulin has been omitted.	

A small RCT consisting of 160 ambulatory surgery patients with T2DM sought to answer whether oral hypoglycaemic drugs (OHDs) should be continued in the perioperative period.^14^ Patients were randomised to continue their OHDs or withhold them. They found that perioperative blood glucose levels were significantly lower (mean, 7.7 mmol L^−1^; confidence interval [CI], 7.2–8.1 mmol L^−1^) in the group that continued *vs* the group that discontinued OHDs (mean, 8.7 mmol L^−1^; CI, 8.1–9.3 mmol L^−1^; *P*<0.001),^14^ concluding that perioperative blood glucose levels were significantly better controlled in patients that continued their OHDs. However, this study was limited by its small sample size and that it evaluated only metformin and sulphonylureas (SUs).^14^ The potential side-effects of sodium-glucose co-transporter-2 (SGLT-2) inhibitors were not addressed.

A single-blind multicentre RCT examined the effects of continuing or withholding metformin from T2DM patients undergoing noncardiac surgery (*n*=70). The primary outcome measures were the differences in perioperative blood glucose and lactate between the groups. They found that postoperative blood glucose was similar (8.2 [1.8] in the metformin group *vs* 8.3 [2.3] mmol L^−1^ in the withheld group; *P*=0.95). Furthermore, they found that although preoperative lactate levels were marginally higher in the metformin group (1.5 *vs* 1.2 mmol L^−1^; *P*=0.02), postoperative lactate levels were not significantly different (1.2 *vs* 1.0 mmol L^−1^; *P*=0.18). The authors concluded that the continuation of metformin in this patient cohort does not cause hypoglycaemia, nor does it significantly raise lactate levels.^15^ Therefore, metformin should be continued perioperatively, even in the fasting patient.

Sulphonylureas are insulin secretagogues that have been in use since the 1950s. They function by stimulating insulin release from beta cells in the pancreas by binding to sulphonylurea receptors, thereby causing hypoglycaemia in the fasted patient by increasing insulin secretion.^16^ Therefore, it is widely accepted that this class of medication is withheld on the day of surgery to avoid the risk of hypoglycaemia. We found little recent evidence regarding the perioperative management of sulphonylureas.

A current focus of research into diabetes pertains to perioperative management of patients with T2DM using medications other than insulin. One small RCT (*n*=90) demonstrated that the glucagon-like peptide-1 receptor agonist (GLP-1RA) liraglutide is superior to insulin in the perioperative management of patients with T2DM undergoing elective surgery within Enhanced Recovery After Surgery (ERAS) protocols. This study showed that the patient cohort receiving liraglutide demonstrated more stable glycaemic levels, required less additional insulin and lower insulin doses on the day of surgery, and less additional insulin volume throughout the perioperative period.^17^

Another small RCT (*n*=70) recruited patients with T2DM undergoing elective cardiac surgery. Patients were randomised to receive either insulin alone or insulin and liraglutide (0.6 mg day^−1^). The primary endpoint was the average *M* value (a derived parameter indicating the proximity of measured blood glucose to the target level) from Day 1 to Day 10 postoperatively. The *M* value in the liraglutide plus insulin group was significantly lower than in the insulin-alone group (liraglutide plus insulin 5.8 [inter-quartile range, IQR=4.4–7.8] *vs* insulin-alone 12.3 [IQR, 9.4–16.0]; *P*<0.001). They concluded that the addition of low dose liraglutide may achieve better glycaemic control in the perioperative period than insulin alone.^18^

A multi-centre open-label RCT (*n*=150) studied the effects of subcutaneous liraglutide in patients with T2DM undergoing noncardiac inpatient surgery. The authors compared three treatment strategies to lower glucose and reduce the need for rescue insulin. One cohort received premedication with liraglutide the night before surgery and the morning of surgery. The other two groups consisted of a glucose–insulin–potassium infusion cohort and an insulin–bolus cohort. The primary outcome was the difference in median glucose levels 1 h after surgery. They found that the median (IQR [range]) plasma glucose at 1 h postoperatively was lower in the liraglutide group (6.6 [5.6–7.7] mmol L^−1^) compared with the insulin infusion group (7.5 [6.4–8.3] mmol L^−1^; *P*=0.026) and insulin bolus groups (7.6 [6.4–8.9] mmol L^−1^; *P*=0.006), respectively.^19^

A multicentre, randomised, blind, placebo-controlled superiority trial in adult patients undergoing cardiac surgery examined the use of subcutaneous liraglutide as an adjunct to improve glycaemic control. The primary endpoint was the difference between groups for any intravenous insulin given in the operating theatre to maintain blood glucose >8–10 mmol L^−1^. They found that (43%) patients from the liraglutide group needed insulin compared with that (61%) in the placebo group – a difference of 18% (95% CI, 5.9–30.0; *P*=0.003).^20^

Another multi-centre RCT (*n*=280) hypothesised that linagliptin (5 mg), a dipeptidyl dipeptidase-4 (DPP-4) inhibitor combined with a fast-acting supplemental insulin, would result in non-inferior glycaemic control compared with a basal-bolus insulin regimen in T2DM patients undergoing noncardiac surgery.^21^ A basal-bolus regimen consists of a combination of a once daily long-acting or intermediate-acting insulin along with very rapid-acting insulin used at mealtime. Usually the basal dose consist of approximately 50% of the total daily insulin dose.^16^ They found that the mean daily blood glucose was higher in the linagliptin group (9.5 [2.3] mmol L^−1^) compared with the basal-bolus group (8.8 [2.3] mmol L^−1^; *P*=0.03). However, patients with linagliptin experienced fewer hypoglycaemic events (1.6% *vs* 11%, *P*=0.001) and needed fewer daily insulin injections (2.0 [3.3] *vs* 3.1 [3.3]; *P*<0.001).^21^

A single-centre RCT examined the use of oral sitagliptin (a DPP-4 inhibitor) in patients with T2DM undergoing coronary artery bypass grafting (*n*=182). The primary outcome was the difference in the proportion of patients with postoperative hyperglycaemia (defined as >10 mmol L^−1^). They noted that the frequency of hyperglycaemia in intensive care postoperatively was not significantly different between groups (75% and 84%, *P*=0.14; difference=–9%; 95% CI, –21%–3%) for patients on sitagliptin and placebo, respectively.^22^

SGLT-2 inhibitors are being increasingly prescribed for patients with T2DM in the community, because of their beneficial cardiorenal properties.^23^ It is expected that, in the near future, the main indication for prescribing SGLT-2 inhibitors will be for cardiorenal protection among DM patients.^23^ Although rare, a concern with SGLT-2 inhibitors is their association with euglycaemic ketoacidosis (euDKA).^24^ It has been suggested that surgery may precipitate euDKA, as the surgical stress response increases ketone production. A retrospective review of 1307 patients on SGLT-2 inhibitors who underwent surgical procedures found that the incidence of euglycaemic diabetic ketoacidosis was 0.2% in non-emergent procedures and 1.1% for emergent procedures, owing to adherence to preoperative instructions to stop the SGLT-2 inhibitor preoperatively in the former group.^25^ Studies are ongoing on the potential cardiorenal protective properties in the perioperative period.

Although guidelines from the Centre for Perioperative Care (CPOC) recommend that SGLT-2 inhibitors are omitted both on the day before surgery and the day of surgery itself,^5^ in contrast, latest guidelines from the European Society of Cardiology (ESC) and European Society of Anaesthesiology and Intensive Care (ESAIC) recommend withholding SGLT-2 inhibitors for 3 days before scheduled surgery, reflecting the increasing concern for euDKA.^26^ This lack of consensus highlights the dearth of clinical research informing these guidelines. Indeed, a consensus statement from the Society for Ambulatory Anaesthesia highlights this: ‘there is insufficient evidence regarding preoperative management of oral antidiabetics’.^27^

Therefore, until more data are available, a conservative approach may be advisable. When a patient using SGLT-2 inhibitors undergoes surgery without interrupting the medication, ketones should be measured in case of ketoacidosis and treatment with insulin by variable rate IV insulin infusion (VRIII) should be initiated intraoperatively to control ketone production. The decision to proceed with the surgery is a risk–benefit estimate, taking the ketone plasma level and other blood gas parameters into account, including pH and base-excess/bicarbonate.

In summary, there seems no clear benefit in continuing metformin or sulphonylureas. The continuation or even initiation of GLP-1 agonists or DPP-4 inhibitors in the perioperative period may be beneficial but pending further studies, SGLT2 inhibitors should be withheld for 2 days among DM patients undergoing elective surgery.

#### Insulin

There are several ways to manage a patient's insulin in the preoperative period (Table 2). The most common are (1) continuation of the patient's normal multiple daily injections of insulin but at a reduced dose before surgery; (2) commencement of a VRIII; (3) continuation of continuous subcutaneous insulin infusion (CSII) – usually via a pump; or (4) fixed rate intravenous insulin infusion (FRIII) – the latter only used for management of diabetic ketoacidosis or hyperosmolar hyperglycaemic non-ketotic (HHONK) state.

The VRIII was advocated as a replacement for Alberti's ‘glucose–insulin–potassium’ (GIK) in the 1990s.^28^ Although Alberti's regimen was a significant improvement in the perioperative management of the patient with diabetes, it had inherent limitations, including high risk of error owing to the number of additives to the fluid bag, and was also wasteful. However, all guidelines caution on the inherent risks of VRIII unless used in a highly monitored environment, such as intraoperatively or in a critical care environment, because of the risk of hypoglycaemia or diabetic ketoacidosis (DKA) if inappropriately managed.^5^ Of note, patients with T1DM always need insulin to avoid the development of DKA.

Guidelines differ somewhat in insulin dose adjustments before surgery for patients using multiple daily injections of insulin and these guidelines are summarised in Table 2. Most guidelines recommend reducing the long-acting insulin dose the day before and the day of surgery 20–30%, depending on the timing of insulin and once or twice daily use. For premixed and short-acting insulin, there are usually no dose adjustments required the day before surgery. On the day of surgery, most guidelines recommend halving the dose for premixed insulins and withholding short-acting insulins without meals (Table 2).

**Fig 1:**
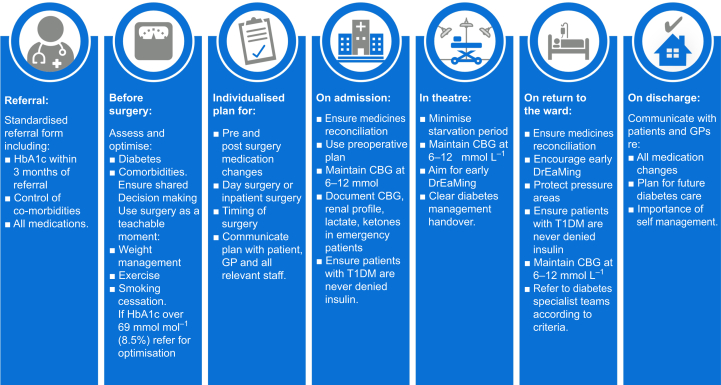
Guideline care pathway for the perioperative management of patients with diabetes mellitus (Reproduced with permission from the Centre for Perioperative Care; https://cpoc.org.uk/sites/cpoc/files/documents/2023-02/CPOC-Diabetes-Guideline-Updated2022_0.pdf; accessed 7 February 2023). CBG, capillary blood glucose; DrEaMing, drinking, eating, and mobilising; HbA1c, haemoglobin A1c; T1DM, type 1 diabetes mellitus.

CSII has been a mainstay of treatment in patients with T1DM for many years and approximately 15% of patients with T1DM use one.^5^ It delivers a fixed hourly rate of a rapid-acting insulin analogue which acts as a patient's basal insulin. To supplement this, patients can give bolus doses of insulin to match their carbohydrate intake.^16^ It offers improved glycaemic control over multiple daily injections of insulin^29^ and a lower risk of hypoglycaemia.^30^ A retrospective observational study demonstrated that patients with T1DM maintain lower haemoglobin A1c (HbA1c) values on CSII over a 1–10-year period compared with pre-CSII values.^31^ The CPOC (Fig. 1) recommends that patients continue their CSII in the perioperative period provided that they have a short fasting time (no more than one missed meal).^5^ It is felt that CSII is safer as it avoids the potential risks of VRIII. Of note, most insulin pumps have not been developed for the perioperative setting and dislocation of the pump or interaction with electrocautery should be monitored.^32^ The user manual of most CSII pumps will state that the pump has not been developed for use in the operating theatre. The CPOC guideline has included a section recommending a shared decision-making risk–benefit discussion with the patient for the perioperative use of CSII (Fig. 1).

As part of the preoperative workup for a patient with T1DM using a CSII pump, it is recommended that a basal test is performed a few days or weeks before surgery.^33^ This means observing the rate of insulin infusion which is necessary to maintain blood glucose 5–10 mmol L^−1^ on the CSII, while the patient is fasting. This allows the patient and the medical team to establish the correct basal infusion rate in the fasted state. Some guidelines recommend continuing the CSII basal rate at 80% of normal whereas others recommend continuing it as normal to counteract the hyperglycaemia that results from the stress response to surgery.^34^

A recent single-centre, open-label, RCT compared the use of closed-loop subcutaneous insulin delivery devices with standard insulin therapy in insulin-requiring patients in the perioperative period undergoing elective surgery. A closed-loop subcutaneous insulin delivery device incorporates a continuous glucose monitoring device which communicates real-time glucose data to the CSII which autonomously delivers insulin to maintain blood glucose within a desired range. Patients were randomised to a closed-loop group or a control group who received standard insulin management as per local policy. The primary endpoint was the proportion of time in which sensor glucose was in the target range of 5.6–10.0 mmol L^−1^. The study found that the mean proportion of time that the sensor glucose was in the target range was 76% (10%) in the closed-loop group and 55% (21%) in the control group (mean difference=22.0 percentage points; 95% CI, 12–32%; *P*=0.001). There were no episodes of hypoglycaemia in either group. The study concluded that, in insulin-requiring patients undergoing surgery, closed-loop subcutaneous insulin delivery devices improve glycaemic control with no increase in risk of hypoglycaemia.^35^

#### Fasting

There are several studies which examine the optimal fasting times for the patient with diabetes. This is of particular interest as there is a risk of gastroparesis, which is often undiagnosed.^36^ Prospective cohort studies showed that the prevalence of incomplete gastric emptying, measured by ultrasound, was higher in the patient with diabetes (48% *vs* 8%, *P*=0.001) compared with the patient without diabetes, despite adherence to local fasting guidelines.^37^ Another study observed that the prevalence of full stomach was 5% (95% CI, 2–9%) in elective patients and 56% (95% CI, 50–62%) in emergency patients (*P*<0.0001), with DM as an independent factor predictive of a full stomach (odds ratio [95% CI], 2.3 [1.2–4.6]; *P*=0.012).^38^

There is also an association between the severity of gastrointestinal symptoms and the level of glycaemic control.^39^ A scoping review notes that the ‘true risk of aspiration in fasting patients with diabetes is unknown’.^40^ The review identified that there are few studies addressing the issue of whether the patient with diabetes is at increased risk of gastric aspiration.

### Preoperative optimisation

Most institutions have guidelines in place for the perioperative management of the patient with diabetes. Unfortunately, these are not always adhered to. A region-wide prospective audit which looked at 17 hospitals in the UK found that compliance with national guidelines was poor. For instance, they found that the mean (standard deviation [sd]) fasting time was 12 (4) h. There was a failure to commence a VRIII in 25 patients who missed two or more meals.^41^ Furthermore, only 8% of patients received the recommended substrate fluid (5% glucose in 0.45% saline) alongside VRIII, and although 87% of DM patients were seen in a preoperative assessment clinic, only 71% had their HbA1c recorded.^41^

Audits demonstrate that the preoperative optimisation of patients with diabetes is lacking in elective cases.^41^ Indeed, the optimum care pathway for the patient with diabetes in the perioperative period for emergency surgery continues to create debate. One guideline recommends that the patient with diabetes be placed on a VRIII while awaiting emergency surgery and should have a capillary blood glucose of 6–10 mmol L^−1^ on arrival to the operating theatre.^8^ A retrospective observational study (*n*=48) observed that a HbA1c level taken on the day of emergency surgery was consistent with patients' pre-morbid HbA1c levels – despite the stress response and inflammation associated with emergency surgery.^42^

A *post-hoc* observational analysis of the Surgical Site Infection (SSI) Trial reported that there is a significant population of patients who have undiagnosed DM, based on their HbA1c levels. They noted that 65% (*n*=45) of a preoperative patient group (*n*=69) who self-reported no diabetes did in fact have HbA1c levels >42 mmol mol^−1^, consistent with pre-diabetes or T2DM. Furthermore, they noted that this cohort had the highest percentage of infections (39%) after major surgery.^43^

These studies highlight the association between preoperative glycaemic control and postoperative outcome. Although preoperative glycaemic optimisation (e.g. HbA1c lowering) seems sensible, studies on the feasibility and efficacy are lacking and needed to substantiate this recommendation.

### Perioperative management

#### Glucose control

There principal objective for the anaesthetist caring for the patient with diabetes is to prevent hyperglycaemia and hypoglycaemia, maintaining blood glucose in the range 5–10 mmol L^−1^. Glucose usually peaks early after surgery, and this postoperative hyperglycaemia is a clear risk factor for complications. In a large retrospective cohort study (*n*=11 633), examining the relationship between perioperative hyperglycaemia and insulin administration on outcomes in elective colorectal and bariatric surgery, patients with hyperglycaemia perioperatively had a significantly increased risk of postoperative infection (odds ratio [OR]=2.0; 95% CI, 1.63–2.44), whether or not there was a pre-existing diagnosis of diabetes.^44^ This is further emphasised by a multicentre prospective observational study (*n*=224) which noted that severe intraoperative hyperglycaemia (>10 mmol L^−1^) is independently associated with new-onset infections in patients undergoing craniotomy, either elective and emergency (OR [95% CI]: 4.2 [1.5–11.5]; *P*=0.006).^45^

The CPOC guidelines recommend that blood glucose is maintained at 6–12 mmol L^−1^.^5^

There are conflicting data regarding the optimal treatment of the patient with diabetes in the perioperative period. One randomised multicentre trial of T2DM patients undergoing elective or emergency surgery demonstrated that a basal-bolus insulin regimen is superior to an insulin sliding scale (mean daily blood glucose concentration of 8.7 [1.8] *vs* 9.8 [2.4] mmol L^−1^; *P*=0.001) in the perioperative management of glycaemic control, also showing a reduction of postoperative complications.^46^ However, it has been demonstrated in other studies that over-zealous control of blood glucose can lead to harmful outcomes such as hypoglycaemia and increased morbidity and mortality.^47^^,^^48^ This must be balanced with data that higher blood glucose levels (>10 mmol L^−1^) increase the risk of postoperative nosocomial infection. A prospective observational analysis noted that a serum glucose level >12.2 mmol L^−1^ on Day 1 postoperatively was a sensitive (87.5%) but relatively non-specific (33.3%) predictor of the development of postoperative nosocomial infection.^49^

An RCT consisting of patients with (*n*=152) and without (*n*=150) diabetes undergoing coronary artery bypass graft (CABG) surgery evaluated the optimal level of glycaemic control to improve outcomes in cardiac surgery patients. Patients with hyperglycaemia were randomised into two groups – an intensive glucose control (target glucose 5.6–7.8 mmol L^−1^ [*n*=151] or a more conservative target of 7.8–10.0 mmol L^−1^ [*n*=151]). The primary outcome was a composite of complications including mortality, wound infection, pneumonia, bacteraemia, respiratory failure, acute kidney injury, or major cardiovascular events. Although median blood glucose was lower 7.3 (IQR, 6.9–7.7 mmol L^−1^) in the intensive control group *vs* 8.6 (0.9) mmol L^−1^ (IQR, 7.9–9.1 mmol L^−1^) in the conservative group (*P*=0.001), no significant difference in the composite outcome was observed (42 *vs* 52%, *P*=0.08).^50^

A meta-analysis which examined the association between hyperglycaemia and SSI found a significant benefit for an intensive glucose control protocol *vs* a conventional protocol in SSIs (OR=0.43; 95% CI, 0.29–0.64; *P*=0.001). Unsurprisingly, there was a significantly higher risk of hypoglycaemic events in the intensive group compared with the conventional group (OR=5.6; 95% CI, 2.6–11.9). They concluded that blood glucose <8.3 mmol L^−1^ reduced the risk of SSI.^51^ A *post-hoc* cost analysis of the Intensive *versus* Conservative Glucose Control in Patients Undergoing Coronary Artery Bypass Graft Surgery (GLUCO-CABG) trial examined the financial cost of intensive (5.5–7.8 mmol L^−1^) *vs* conservative (7.9–10 mmol L^−1^) blood glucose control in the ICU in patients with and without diabetes undergoing CABG. They found that median hospitalisation costs were lower in the intensive group ($39 366 *vs* $42 141, *P*=0.040) than in the conservative group.^52^ Data from ICU studies show that hyperglycaemia may be more harmful in patients with lower admission HbA1c,^53^ suggesting that future perioperative studies could explore personalised glucose targets.

A small single-centre prospective observational study (*n*=52) consisting of patients with diabetes undergoing open nephrectomy surgery evaluated the association between long-term glycaemic control and postoperative analgesic requirements. Patients were divided into two cohorts – those with good glycaemic control (HbA1c <6.5%) and those with poor glycaemic control (HbA1c ≥6.5%). They found that, in the first 48 h postoperatively, fentanyl consumption was 20% higher in the cohort with poor glycaemic control (*P*=0.007). This cohort also reported higher rates of inadequate analgesia (89% *vs* 67% on movement, *P*=0.03), suggesting that preoperative HbA1c level may be useful in anticipating postoperative analgesic requirements.^54^ Intermediate (up to 30 days) and longer-term outcomes in the postoperative patient with diabetes are not well researched. A small RCT (*n*=151) examined the effect of continued follow-up care by a hospital diabetes team on HbA1c levels 1 yr after discharge in patients with diabetes who underwent elective surgery. Patients were randomised into a continued care (CC) group or a usual care (UC) group. The research team found that the HbA1c at 1 yr in the continued group was 8.2 (1.4) *vs* 8.5 (1.5) in the UC group (*P*=NS), indicating that continued follow-up by a hospital diabetes team did not have an additional impact on long-term glycaemic control; however, this study may have been underpowered.^55^

However, in terms of postoperative care, research is again scarce, and evidence mostly comes from small, underpowered studies. The ideal glucose target is not yet clearly elucidated. The adequate guidance and treatment of patients with diabetes in the postoperative period eagerly awaits larger trials. Personalised glucose targets, taking account of a given patient's preoperative control, co-morbidities, and predilection to hypo- or hyperglycaemia, may be more appropriate.

### Adjuvant drugs and impact on glucose control

#### Dexamethasone

A randomised stratified multi-centre trial examined the effects of dexamethasone on perioperative blood glucose levels (PADDAG trial). They evaluated patients (*n*=302) undergoing general anaesthesia for elective noncardiac surgery who received either intravenous placebo, 4 or 8 mg of dexamethasone. Patients were stratified into non-diabetic, T1DM, and T2DM. The primary outcome was the perioperative blood glucose profile up to 24 h after surgery. They found that a single dose of dexamethasone (4 or 8 mg) did not significantly affect the maximal blood glucose in the 24-h period after surgery, 10.3 (8.1–12.4), 12.6 (10.3–18.3), and 13.6 (11.2–20.1) mmol L^−1^ in the placebo, dexamethasone 4 mg, and dexamethasone 8 mg groups, respectively (*P*=0.15).^56^ In contrast, a retrospective study (*n*=1037) compared the change in postoperative blood glucose from preoperative values in patients with T2DM after elective surgery who received 4, 8, or 10 mg of dexamethasone for the prophylaxis of postoperative nausea and vomiting (PONV). They found that the patients who received 8 or 10 mg had a greater increase in blood glucose level compared with the 4 mg dose in the PACU (mean [sd], 3.2 [2.8] *vs* 2.4 [2.5] mmol L^−1^; *P*<0.0001) over 24 h. Higher doses of dexamethasone are associated with greater perioperative increase in blood glucose levels compared with a 4 mg dose.^57^

A definitive multi-centre, randomised, controlled, non-inferior trial examined the effects of perioperative intravenous dexamethasone administration on postoperative surgical site infection rates in patients undergoing elective noncardiac surgery (Perioperative Administration of Dexamethasone and Infection [PADDI] trial).^58^ Their primary endpoint was the occurrence of SSI within 30 days of the day of surgery. More than 8880 patients were randomised to receive either intravenous dexamethasone 8 mg or placebo during anaesthesia. Of this group, 13% had diabetes. SSI occurred in 8.1% in the dexamethasone group and 9.1% in the placebo group (risk difference adjusted for diabetes status, –0.9 percentage points; 95.6% CI, –2.1 to 0.3; *P*<0.001 for non-inferiority). Of note, PONV in the first 24 h after surgery occurred in 42% in dexamethasone and in 54% in placebo (risk ratio=0.78; 95% CI, 0.75–0.82).^59^

It is important in the patient with diabetes that normal diet is resumed as soon as possible. The occurrence of PONV can delay resumption of normal eating and drinking which, in the patient with diabetes, can lead to further derangement of blood glucose control, increased risk of ketoacidosis and duration of insulin infusions, and delayed hospital discharge.

#### Magnesium sulphate

It is hypothesised that magnesium decreases blood glucose through several mechanisms – increasing the affinity of insulin to its receptors, increasing secretion of insulin by the pancreas, potentiating insulin mediated glucose uptake, regulation of glycogenolysis and glucose output in the liver, decreasing release of catabolic hormones, and regulating glucose translocation into the cell.^5^

A double-blinded RCT (*n*=122) examined the efficacy of magnesium sulphate in reducing the blood glucose in diabetic patients undergoing cardiac surgery. The intervention group received a continuous infusion of magnesium sulphate at 15 mg kg^−1^ h^−1^ which was commenced 20 min before induction and continued for the first 24 h postoperatively. The control group received normal saline. They found that blood glucose and insulin requirements decreased in the magnesium patients. At ICU admission postoperatively, the patients receiving magnesium had a mean blood glucose of 8.8 (1.5) *vs* 9.7 (1.5) mmol L^−1^ in the control group (*P*=0.003).^60^

## Regional anaesthesia

It can be beneficial to consider regional anaesthesia in the patient with diabetes mellitus. Some of the benefits include the avoidance of airway complications, reduced incidence of PONV, earlier resumption of diet, decreased duration on insulin infusions, earlier mobilisation, opioid sparing, and reduced length of stay.^61^ Reviews have concluded that patients with diabetes have an increased risk for the occurrence of difficult intubation – the most important contributing factors were found to be obesity, increased neck circumference, and stiff joint syndrome.^62^^,^^63^

A small prospective RCT (*n*=68) examined the effect of spinal anaesthesia *vs* general anaesthesia on the surgical stress response and perioperative hyperglycaemia in patients with and without diabetes mellitus undergoing elective total hip replacement. A significant increase in glucose levels in both diabetic and non-diabetic patients who underwent a general anaesthetic compared with spinal anaesthesia was observed. They concluded that spinal anaesthesia attenuates the hyperglycaemic response to surgical stimuli both in patients with and without diabetes mellitus.^64^

Some differences for patients with diabetes as compared with patients without diabetes may apply and are relevant for both the patient and anaesthetist, including longer block duration, a theoretical increased risk of neuropathy, and – when using a catheter – a possible increased infection risk.

## Preventable harms

Whenever possible, hospital admission for patients with diabetes mellitus should be limited. The NHS National Diabetes Inpatient Audit (NaDIA) Harms 2020 reported 4605 serious events with harm for patients with diabetes mellitus. The most common event was hypoglycaemia, followed by in-hospital DKA, foot ulcer, and hyperosmolar hyperglycaemic state (HHS). Recommendations from the NaDIA included participation in the Getting It Right First Time (GIRFT) program to optimise the surgical pathway, proper identification, and referral of patients with diabetes mellitus (including type) and aiming for avoidance of DKA and HHS.^65^

Limiting hospital admission, for example by ambulatory surgical treatment, has the potential of reducing iatrogenic harm, such as insulin administration errors, as described in the GIRFT diabetes workstream. Standardised perioperative care pathways have proven beneficial for patients with diabetes mellitus, although it should be noted that diabetes mellitus remains a risk factor for re-admission after ambulatory surgery.^66^

## Future research

One of the challenges facing future research of this topic is the lack of consistency in defining diabetes mellitus itself.^10^ This makes it difficult to identify trends, associations, and perioperative outcomes in this large cohort of patients. It is also evident that many of the available recommendations and guidelines are written based on consensus and expert opinions rather than on Level I or Level II clinical evidence. Furthermore, medications available to patients with DM are constantly evolving with new types of medications, especially GLP-1 agonists, DPP-4 inhibitors, and SGLT-2 antagonists; therefore, there is a lack of available clinical data evaluating these to optimise the perioperative course of the diabetic patient. Next to new drugs, evolving wearable technology may also be of use for diabetes patients in the in-hospital setting, especially continuous glucose sensors, which could contribute to discharging patients to a virtual ward. It is possible that personalised treatment targets and the perioperative application of new antidiabetic medicines will be the study focus the coming years. The ongoing ‘Management and outcomes of the perioperative care of European diabetic patients’ (MOPED) study will provide the largest prospective observational data on the perioperative journey of patients with diabetes mellitus, up to 30 days after undergoing surgery.^67^ This may generate hypotheses for RCTs on whether patients with poorly controlled DM preoperatively benefit from increased glycaemic control postoperatively, and whether any particular anaesthetic technique or perioperative glycaemic management favours preferred postoperative outcomes. It is likely that coordinated research networks focused on optimising perioperative management of patients with diabetes mellitus will be needed to generate sufficient power in clinical RCTs to inform best practice.

## Authors' contribution

KC: Literature search and screening for relevant material, drafting manuscript for intellectual content, checking references.

PO'S: Literature search and screening for relevant material, proof-reading manuscript for intellectual content.

JH: Literature search and screening for relevant material, drafting and redrafting manuscript for intellectual content, checking references.

DJB: Instigator and identification of need within existing literature; Literature search and screening for relevant material, drafting and proof-reading manuscript for intellectual content.

## Declaration of interest

None of the other authors have any conflict of interest.
