# Supplementary material for: Current practice in the perioperative management of patients with diabetes mellitus: a narrative review
Source: Br J Anaesth. 2023 Apr 13;131(2):242–52. doi: 10.1016/j.bja.2023.02.039 (PMC10375498; doi:10.1016/j.bja.2023.02.039)
Supplement: Multimedia component 1 [file mmc1.docx]

**Case discussion**

A 44-year-old female is planned for elective ankle surgery because of a malunion of an old fracture. She has a history of diabetes mellitus type 1 (T1DM), which is treated with continuous subcutaneous insulin infusion (CSII), with different basal bolus rates throughout the day, ranging between 0.4-1.4 IU/hour. For meals, she administers boluses from her pump, ranging from 4-8 IU, depending on the meal and her glucose level. Her glucose levels are continuously measured using a continuous subcutaneous glucose meter (CSGM). Both the CSII pump and her CSGM sensor are inserted in her abdomen. She is usually well regulated, with an HbA1c of 50 mmol/mol and no serious hypoglycaemia in the last year.

She has no further medical history, and no diabetes-related organ damage. However, during a previous procedure, her CSII was removed and she did not receive any alternative sc or iv insulin, resulting in a diabetic keto-acidosis, from which she recovered. She insists on keeping her CSII pump and CSGM during and after the procedure.

Even though manufacturers usually advise replacing CSII during surgery, the ‘Guideline for perioperative care for people with diabetes mellitus undergoing elective and emergency surgery’ from the Centre for Perioperative Care (CPOC), has included a section on shared decision making for these specific situations. According to the CPOC protocol, a risk-benefit analysis together with the patient, which was documented in the chart, resulted in using her CSII pump during the surgery. Basal-bolus rates were set at 80% of normal and no additional boluses were administered by the patient on the morning of surgery. She preferred general anaesthesia, and even though her CSGM was reliable in daily life, capillary blood glucose was measured every hour, as CSGM may be unreliable during surgery.

Before and during surgery, the CSII-insertion site was checked and glucose target was 6-10 mmol/l. After surgery, as soon as the patient was awake and alert and able to eat and drink, she resumed control over her glucose regulation herself.
